# Deep learning-based automatic surgical step recognition in intraoperative videos for transanal total mesorectal excision

**DOI:** 10.1007/s00464-021-08381-6

**Published:** 2021-04-06

**Authors:** Daichi Kitaguchi, Nobuyoshi Takeshita, Hiroki Matsuzaki, Hiro Hasegawa, Takahiro Igaki, Tatsuya Oda, Masaaki Ito

**Affiliations:** 1 Surgical Device Innovation Office, National Cancer Center Hospital East, 6-5-1, Kashiwanoha, Kashiwa, Chiba, 277-8577 Japan; 2 Department of Colorectal Surgery, National Cancer Center Hospital East, 6-5-1, Kashiwanoha, Kashiwa, Chiba, 277-8577 Japan; 3 Department of Gastrointestinal and Hepato-Biliary-Pancreatic Surgery, Faculty of Medicine, University of Tsukuba, Tsukuba, Ibaraki 305-8575 Japan

**Keywords:** TaTME, Video dataset, Step classification, Computer vision, Deep learning, Convolutional neural network

## Abstract

**Background:**

Dividing a surgical procedure into a sequence of identifiable and meaningful steps facilitates intraoperative video data acquisition and storage. These efforts are especially valuable for technically challenging procedures that require intraoperative video analysis, such as transanal total mesorectal excision (TaTME); however, manual video indexing is time-consuming. Thus, in this study, we constructed an annotated video dataset for TaTME with surgical step information and evaluated the performance of a deep learning model in recognizing the surgical steps in TaTME.

**Methods:**

This was a single-institutional retrospective feasibility study. All TaTME intraoperative videos were divided into frames. Each frame was manually annotated as one of the following major steps: (1) purse-string closure; (2) full thickness transection of the rectal wall; (3) down-to-up dissection; (4) dissection after rendezvous; and (5) purse-string suture for stapled anastomosis. Steps 3 and 4 were each further classified into four sub-steps, specifically, for dissection of the anterior, posterior, right, and left planes. A convolutional neural network-based deep learning model, Xception, was utilized for the surgical step classification task.

**Results:**

Our dataset containing 50 TaTME videos was randomly divided into two subsets for training and testing with 40 and 10 videos, respectively. The overall accuracy obtained for all classification steps was 93.2%. By contrast, when sub-step classification was included in the performance analysis, a mean accuracy (± standard deviation) of 78% (± 5%), with a maximum accuracy of 85%, was obtained.

**Conclusions:**

To the best of our knowledge, this is the first study based on automatic surgical step classification for TaTME. Our deep learning model self-learned and recognized the classification steps in TaTME videos with high accuracy after training. Thus, our model can be applied to a system for intraoperative guidance or for postoperative video indexing and analysis in TaTME procedures.

**Supplementary Information:**

The online version contains supplementary material available at 10.1007/s00464-021-08381-6.

Transanal total mesorectal excision (TaTME) was introduced in 2010 to address the limitations of conventional transabdominal total mesorectal excision (TME) [1]. To overcome technical difficulties, a transanal endoscopic surgical approach comprising laparoscopic “rendezvous” above the prostate was advocated, especially for obese men [2]. In particular, the transanal approach is aimed at increasing visibility and providing better access to dissection planes during excision, thereby improving the quality of a resected specimen. In addition, TaTME offers a safer anastomotic technique with the use of a distal purse-string suture, thus allowing low anastomosis in patients who might otherwise need a permanent stoma [3–5].

However, during TaTME, surgeons have experienced intraoperative technical difficulties in approximately 40% of the cases; these technical difficulties include inaccurate plane dissection, pelvic bleeding, and visceral injuries [6]. Expert surgeons and early adopters of the TaTME procedure have acknowledged that these technical difficulties are partly due to unfamiliar views and difficulty interpreting the anatomy from below, which could make it hard to recognize correctly the appropriate tissue planes. This is likely to have been the cause of early reports of urethral injuries reported in the TaTME international registry data [7], which are complications rarely observed in the case of conventional TME surgery.

Video-based learning for minimally invasive surgery is considered a useful teaching aid [8, 9], and it is especially valuable in the case of TaTME with the risk of unexpected complications in patients. Consistent review of intraoperative laparoscopic videos could facilitate understanding of common errors during surgery and increase the awareness of potential injury mechanisms by acknowledging error-event patterns [10, 11]. In addition, several studies showed that video-based learning contributed to reducing surgical error and improving surgical skill [12, 13]; however, manual video review by humans is a time-consuming task.

Convolutional neural networks (CNNs) [14] are a type of artificial intelligence (AI) tool that can be utilized in the field of computer vision for deep learning-based image analysis [15]. Notably, CNNs could be used to review surgery videos in order to identify specific segments of a surgery [16–19]. This would make video-based learning for TaTME considerably more efficient by reducing the effort required in manual video indexing.

Thus, in this study, we constructed an annotated video dataset for segments of the TaTME surgical procedure using a deep learning model to promote video-based learning for TaTME. Moreover, we evaluated the performance of the proposed deep learning model for analyzing intraoperative videos to identify different surgical steps during TaTME.

## Materials and methods

### Study design and patient cohort

This was a single-institutional retrospective feasibility study. Intraoperative video data for 50 patients who underwent TaTME at the Department of Colorectal Surgery at National Cancer Center Hospital East (Kashiwa, Japan) between May 2018 and July 2019 were randomly extracted for the study. However, intraoperative video data for cases wherein the perineal procedure was not properly recorded were excluded from this study.

### Video dataset

In the video dataset, all perineal procedures of TaTME were performed laparoscopically, instead of robotically, and five attending colorectal surgeons performed the procedures. Among the five surgeons, one was a TaTME expert, three had performed 10–30 TaTME surgeries, and the remaining surgeon had performed less than 10 TaTME surgeries.

During preprocessing, the intraoperative TaTME videos were converted to MP4 video format with a display resolution of 1280 × 720 pixels and a frame rate of 30 frames per second (fps). After preprocessing, the video dataset was divided into training and testing sets with 80% and 20% of the data, respectively (i.e., 40 videos were utilized to train models, while 10 videos were utilized to test them). The data were split on a per-video rather than a per-frame basis; thus, frames from a video that were included in the training set were not present in the test set.

### Annotation of surgical steps

The surgical steps of TaTME for annotation in intraoperative videos were determined based on a previous study by Lacy et al. wherein the stepwise procedure for TaTME is described [3, 20]. Given the nature of supervised deep learning, it is considered reasonable to define the surgical steps for the automatic classification task based on the stylized stepwise procedure. Each intraoperative video was manually annotated at 30 fps and parts of the video were manually classified into the following major steps: (1) purse-string closure; (2) full thickness transection of rectal wall; (3) down-to-up dissection; (4) dissection after rendezvous; and (5) purse-string suture for single stapling technique (SST). Steps 3 and 4 were each further classified into four sub-steps; specifically, dissection for anterior, posterior, and both bilateral planes. In this study, the areas of neurovascular bundle and pelvic splanchnic nerves were considered to be a part of the bilateral planes. Every annotation label was manually assigned by two colorectal surgeons (DK and TI) independently, and both surgeons underwent sufficient annotation training and had sufficient knowledge of TaTME. Every discrepancy about the annotation label was solved via discussion. Details on each step including the definitions of the start and end of a step are summarized in Table 1.

**Table 1:** Intraoperative surgical steps and sub-steps during a TaTME

	Surgical step/Sub-step during TaTME	Definitions of start and end of step
1	Purse-string closure	Start: Appearance of suture on screen End: Disappearance of suture from screen
2	Full thickness transection of rectal wall	Start: Approach to rectal wall for cutting End: Completion of transection
3	Down-to-up dissection	
3-1	Anterior plane	Start: Approach to each plane for dissection End: Withdrawal from each plane
3-2	Posterior plane	
3-3	Right plane	
3-4	Left plane	
4	Dissection after rendezvous	
4-1	Anterior plane	Start: Approach to each plane for dissection End: Withdrawal from each plane
4-2	Posterior plane	
4-3	Right plane	
4-4	Left plane	
5	Purse-string suture for SST	Start: appearance of suture on screen End: disappearance of suture from screen

*TaTME* transanal total mesorectal excision, *SST* single stapling technique

### CNN model

In this study, a CNN model, Xception [21], was used for the TaTME surgical step classification task. The model was pre-trained using the ImageNet dataset, which consists of 14 million images of general objects, such as animals, scenes (e.g., beaches, mountains), and food [22]. Data augmentation was not performed.

### Computer specifications

All modeling procedures were performed using a script written in Python 3.6. Furthermore, a computer equipped with an NVIDIA Quadro GP 100 GPU with 16 GB of VRAM (NVIDIA, Santa Clara, CA) and an Intel® Xeon® CPU E5-1620 v4 @ 3.50 GHz with 32 GB of RAM were utilized for model training and testing.

### Evaluation metrics

To evaluate the performance of the CNN model in the surgical step classification task, precision, recall, F1 score, and overall accuracy were measured. The following calculation formulas were used for these metrics.

$$ {\text{Precision}} = \frac{{{\text{TP}}}}{{({\text{TP}} + {\text{FP}})}} $$

$$ {\text{Recall}} = \frac{{{\text{TP}}}}{{({\text{TP}} + {\text{FN}})}} $$

$$ {\text{F}}1\;{\text{score}} = 2 \times \frac{{{\text{Precision}} \times {\text{Recall}}}}{{{\text{Precision}} + {\text{Recall}}}} $$

$$ {\text{Overall}}\;{\text{accuracy}} = \frac{{({\text{TP}} + {\text{TN}})}}{{({\text{TP}} + {\text{FP}} + {\text{FN}} + {\text{TN}})}} $$

where TP, FP, FN, and TN denote true-positive, false-positive, false-negative, and true-negative cases, respectively. Notably, precision, recall, and F1 scores were utilized as performance metrics for each surgical step, whereas overall accuracy was utilized as the performance metric for the entire model. Descriptions of the evaluation metrics are provided in Table 2. Cross-validation was not performed.

**Table 2:** Descriptions of evaluation metrics

Evaluation metrics	Description
True-positive	Number of frames whose predicted step is Step X when the true step is also Step X. (Correct)
False-positive	Number of frames whose predicted step is Step X when the true step is not Step X. (Misclassification)
False-negative	Number of frames whose predicted step is not Step X when the true step is Step X. (Misclassification)
True-negative	Number of frames whose predicted step is not Step X when the true step is also not Step X. (Correct)
Precision	Proportion of correct predictions in all frames predicted as Step X. (Positive predictive value)
Recall	Proportion of correct predictions in each surgical step. (Sensitivity)
F1 score	Harmonic mean of the precision and recall in each surgical step when the concept of true-negative is excluded.
Overall accuracy	Proportion of correct predictions in all frames.

### Institutional approval

The protocol for this study was reviewed and approved by the Ethics Committee of the National Cancer Center Hospital East (Registration No.: 2018–100). This study conforms to the provisions of the Declaration of Helsinki 1964 (as revised in Brazil in 2013).

## Results

### Video dataset

**Fig. 1:**
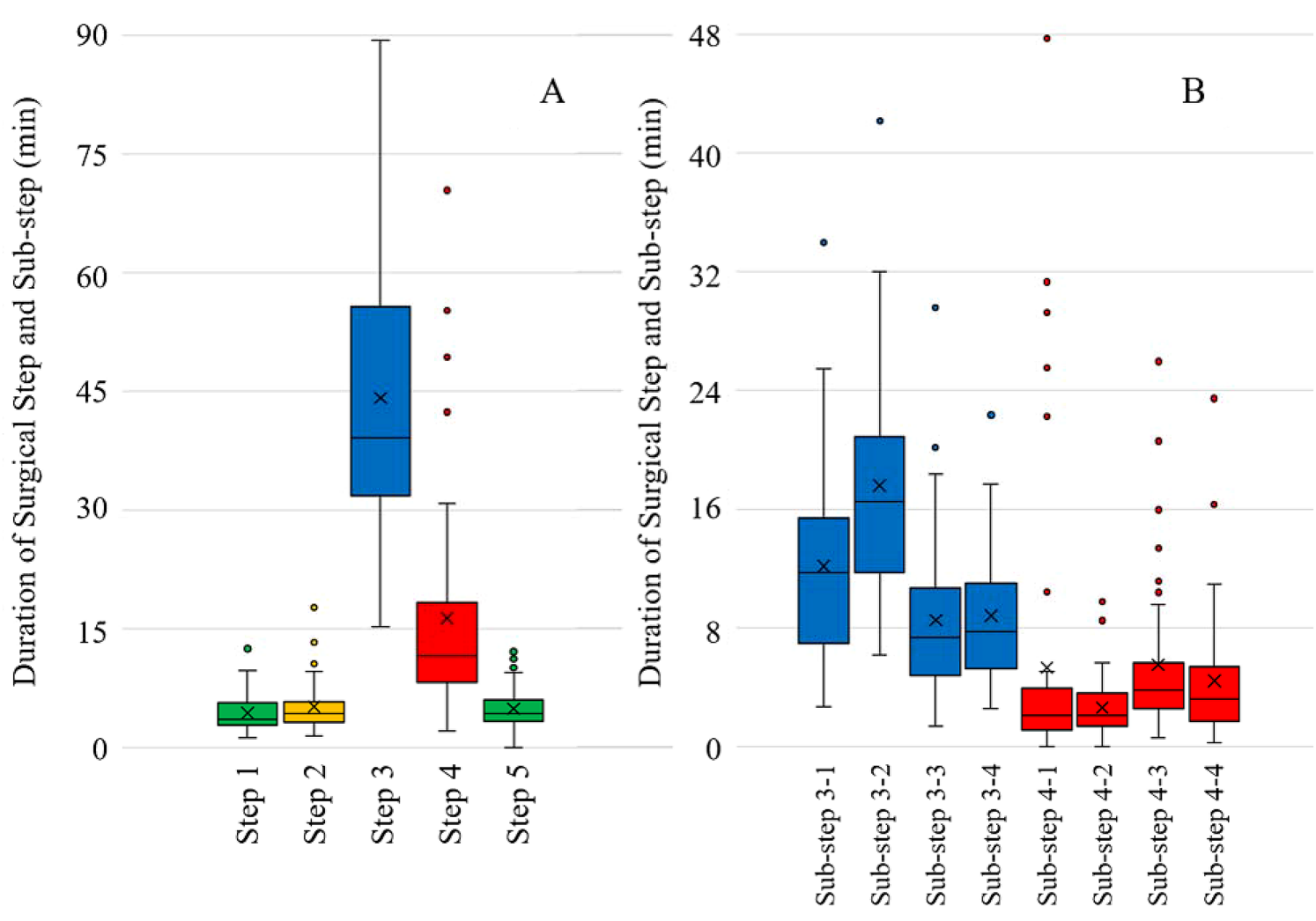
**A** Duration of each surgical step and variation between different cases. **B** Duration of each dissection sub-step and variation between different cases. The duration for sub-step 3-2 (down-to-up dissection on the posterior plane) was the longest on average (16 ± 6.5 min), whereas that for sub-step 4-2 (posterior dissection after rendezvous) was the shortest (2.5 ± 2 min). (green: surgical step related to purse-string suture; yellow: rectotomy step; blue: dissection step before rendezvous; red: dissection step after rendezvous) (Color figure online)

**Fig. 2:**
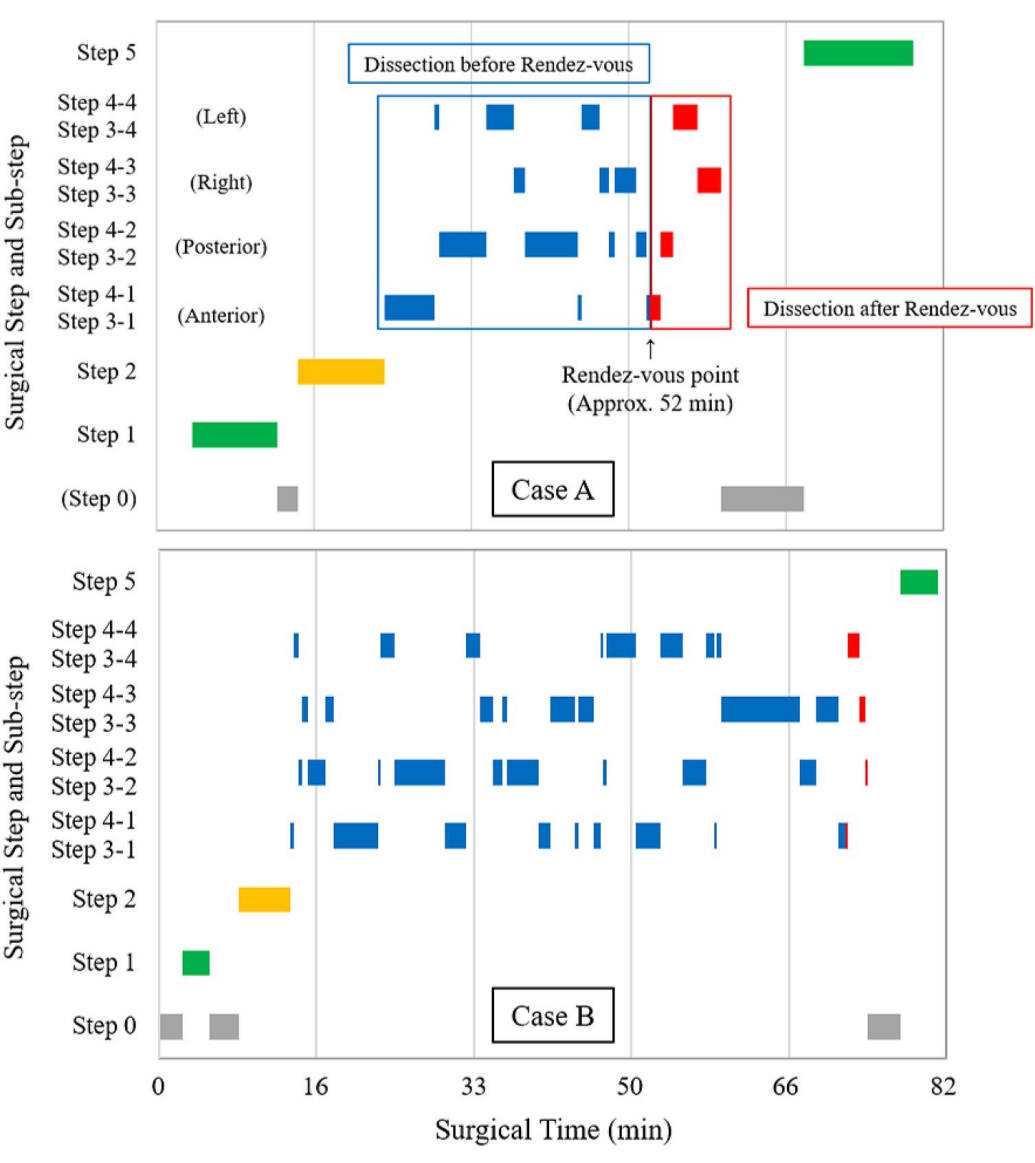
Trace of surgical steps for two representative TaTME cases (green: surgical step related to purse-string suture; yellow: rectotomy step; blue: dissection step before rendezvous; red: dissection step after rendezvous; gray: extracorporeal step) (Color figure online)

Fifty patients were included in the study cohort, of which 30 were men. The median age was 64 years (range 33–83 years), and the median body mass index was 22 kg/m^2^ (range 15–30 kg/m^2^). In terms of preoperative diagnosis, rectal adenocarcinoma was observed in 42 cases, neuroendocrine tumors in five cases, and gastrointestinal stromal tumors in three cases. The most common clinical stage was I (31 out of 42). Furthermore, anastomosis was performed via SST in 43 cases with the median anastomotic height from the anal verge being 5 cm with a range of 1–8 cm. The overall procedure operative time of TaTME was 188 min (with a standard deviation of 60 min), and the average total time for the five major steps in a TaTME was 71.5 min (with a standard deviation of 20.5 min); however, the duration of the individual surgical steps varied for different cases (Fig. 1). Step 5 (i.e., purse-string suture for SST) was not annotated in six cases, because hand-sewn anastomosis was performed in those cases. In the dissection steps (i.e., Steps 3 and 4), sub-step transitions (i.e., transitions between each dissection plane during TaTME) occurred 27 ± 8 times. Rendezvous occurred 29 and 16 times out of 50 on the anterior and posterior sides, respectively. A trace of the surgical steps during two representative cases is shown in Fig. 2. In the figure, case A has a duration of 80 min with 22 surgical step transitions wherein rendezvous occurs on the anterior side at approximately 52 min.

The characteristics of patients whose intraoperative videos formed the training and test sets of the video dataset used in this work are summarized in Table 3. As can be observed in the table, there were no statistically significant differences in patients’ characteristics between the data subsets.

**Table 3:** Characteristics of patients in the study cohort

Characteristics	Training set (*N* = 40)	Test set (*N* = 10)	*P-*value
Sex (male)	23 (58%)	7 (70%)	0.720
Age (years)	66 [41–83]^a^	68 [33–78]^a^	0.913
BMI (kg/m^2^)	22 [15–30]^a^	20 [15–27]^a^	0.0787
Preoperative diagnosis			1.00
Rectal adenocarcinoma	33 (83%)	9 (90%)	
Rectal NET	4 (10%)	1 (10%)	
Rectal GIST	3 (8%)	0	
Clinical stage of carcinoma cases (UICC 8th edition)			0.308
I	25 (76%)	6 (67%)	
II	4 (12%)	3 (33%)	
III	4 (12%)	0	
IV	0	0	
Tumor lower edge from AV (cm)	7 [3–10]^a^	8 [5–10]^a^	0.0952
Abdominal approach			
Laparoscopy	40 (100%)	10 (100%)	-
Robot	0	0	
Open	0	0	
Anastomotic type			0.319
SST	33 (88%)	10 (100%)	
Hand-sewn	7 (12%)	0	
Anastomotic height			
From AV (cm)	5 [1–8]^a^	6 [3–7]^a^	0.0767
From anorectal ring (cm)	1 [−3 to 4]^a^	2 [0–3]^a^	0.0909

*BMI* body mass index, *NET* neuroendocrine tumor, *GIST* gastrointestinal stromal tumor, *UICC* Union for International Cancer Control, *AV* anal verge, *SST* single stapling technique

^a^Median [range]

### Surgical step classification

**Fig. 3:**
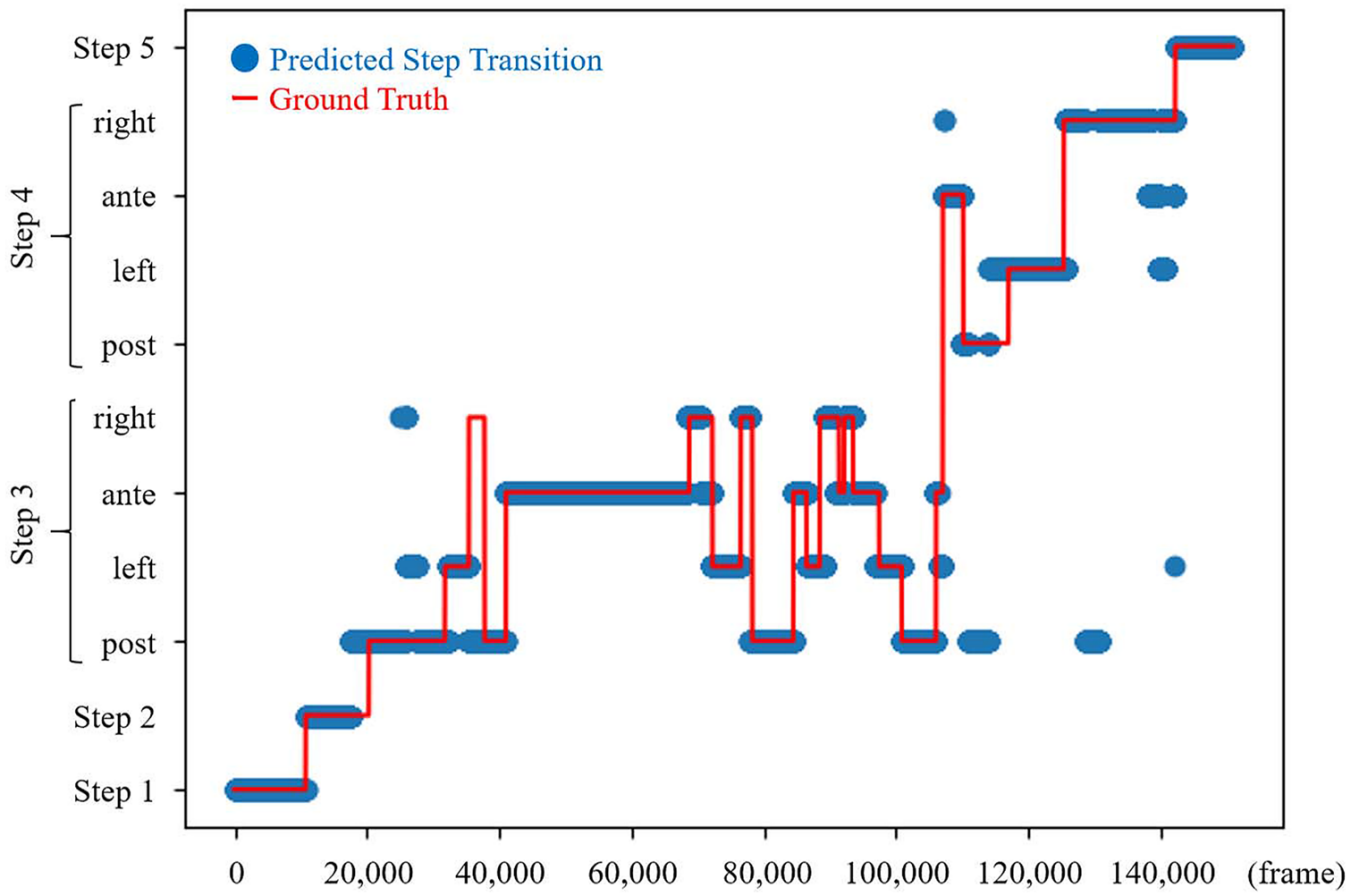
Result of a surgical step classification in a representative TaTME case (blue: predicted step transitions; red: true step transitions) (Color figure online)

Precision, recall, and F1 score for each surgical step and overall accuracy metrics for the entire model are listed in Table 4. The overall accuracy for classification of all the five major steps was 93.2%. However, when sub-step classification was included in the calculation of the performance metrics, the overall accuracy deteriorated to 76.7%, and the mean accuracy of the model for classification of the 11 steps including sub-steps for 10 cases in the test dataset was 78 ± 5% with a maximum accuracy of 85% (Table 5). The results for surgical step classification in a representative case are shown in Figure 3, and the confusion matrix of the results for surgical step classification is shown in Supplementary Appendix A.

**Table 4:** Precision, recall, and F1 score of each surgical step and overall accuracy of the entire model

Surgical step	Precision	Recall	F1 score
Step 1	0.99	0.82	0.90
Step 2	0.83	0.62	0.71
Step 3	0.91	0.99	0.95
Step 4	1.00	0.88	0.94
Step 5	0.99	1.00	0.99
Overall accuracy: 93.2%

**Table 5:** Precision, recall, and F1 score of 11 surgical steps, including sub-steps, and overall accuracy of the entire model when sub-step classification was included in the calculation of the performance metrics

Surgical step	Precision	Recall	F1 score
Step 1	0.99	0.84	0.91
Step 2	0.85	0.75	0.80
Sub-step 3-1	0.86	0.80	0.83
Sub-step 3-2	0.77	0.68	0.72
Sub-step 3-3	0.76	0.84	0.80
Sub-step 3-4	0.54	0.64	0.58
Sub-step 4-1	0.53	0.75	0.62
Sub-step 4-2	0.63	0.80	0.70
Sub-step 4-3	0.71	0.49	0.58
Sub-step 4-4	0.64	0.75	0.69
Step 5	0.98	1.00	0.99
Overall accuracy: 76.7%

## Discussion

In this study, we demonstrated that our deep learning model could recognize the surgical steps of TaTME with a high degree of accuracy (93.2%). This result suggests that an AI-based model can self-learn, analyze, and index TaTME videos on behalf of humans.

In recent years, the use of AI in surgery has attracted significant attention from researchers. Although the use of AI-based methods has its challenges, these methods can improve surgical procedures in the operating room via different approaches [23], including preoperative planning [24, 25], intraoperative guidance [26], and their integrated use in surgical robotics [27, 28]. Annotated datasets are the foundation for several AI-based approaches; however, the complexity of surgery renders the interpretation and management of large amounts of intraoperative video data difficult. Thus, dividing a surgical procedure into a sequence of identifiable and meaningful steps can aid in data acquisition, storage, and analysis, among others.

Thus far, most studies related to surgical step recognition modeling have focused on laparoscopic cholecystectomy because of its standard and frequent execution [16, 29–31]. However, recently, to improve step recognition systems and extend their range of applications, increasingly diverse and complex procedures have been subjected to step recognition modeling, including laparoscopic total hysterectomy [32], robot-assisted partial nephrectomy [17], laparoscopic sleeve gastrectomy [18], and laparoscopic colorectal surgery [19]. Nevertheless, to the best of our knowledge, this is the first study based on the automatic surgical step classification task for TaTME.

Because TaTME is a complex procedure and requires specialized knowledge of pelvic anatomy, which is an unfamiliar topic for many surgeons, safe implementation of TaTME requires surgeons to undergo systematic and structured training [33]; therefore, surgical trainers consider video-based learning to be a useful teaching aid to maximize learning. The automatic surgical step classification for TaTME using a CNN-based approach is a challenging task for the following reasons. First, the quality of intraoperative images is often poor because of an unstable pneumopelvis due to excessive smoking. Second, because the intraoperative field and instruments are seldom changed during TaTME compared with those during other laparoscopic abdominal surgeries, it is difficult to distinguish between different steps, especially sub-steps during dissection. However, this challenging task to classify the plane of dissection (anterior, posterior, or lateral plane) during TaTME should be tackled to develop a quick video dataset indexing system to make video-based learning for TaTME considerably more efficient.

In this study, we constructed the first annotated video dataset for TaTME. The initial purpose of this dataset construction was training and testing of our deep learning model. However, we observed significant differences among different intraoperative videos in terms of step duration, order of sub-steps, and frequency of sub-step transitions by analyzing the annotated dataset. As an example, progressions of surgical steps during two representative TaTME procedures are shown in Fig. 2. In the figure, although the total surgical times in both cases A and B were almost equivalent (80 and 82.5 min, respectively), the duration of each step, order of sub-steps, and frequency of sub-step transitions (17 and 38, respectively) were significantly different. In a future study, we will attempt to obtain correlations between novel parameters, skills, or intraoperative complications, using detailed analyses on a larger dataset, which could then be applied for skill assessment or complication prediction.

This study has several limitations. First, cross-validation was omitted in this study because there were no statistically significant statistical differences in patients’ characteristics between the training and test sets (Table 3) and because we considered the number of frames in the dataset to be sufficiently large (> 650,000 frames); however, the number of analyzed procedures (*n* = 50) and surgeons performing them (*n* = 5) was limited. Therefore, considering the impact of a possible imbalance between the training and test sets (procedure techniques, anatomy, surgeon skill, and learning curve), cross-validation might have been more appropriate. With regard to validation methods, the most appropriate one for each situation should always be considered. Second, the videos that form our dataset were obtained from one institution; thus, the complexity of the data is limited to case variability. Training a deep learning model with such a dataset can lead to over-fitting, which could subsequently reduce the generalizability of the network. To obtain more generalized networks, videos from other medical institutions should be included to ensure higher variability in the dataset. Third, although the accuracy for classification of the five defined major steps was high, there was still room for improvement in the accuracy of classification when sub-steps were included in performance analysis. The difference between the two results could be attributed to the following: first, the fewer the steps to classify, the easier the task would be, and second, although the image features differed significantly between each major step (e.g., purse-string closure vs down-to-up dissection), the differences in image features between each sub-step (e.g., anterior vs right plane dissection) were too slight to classify accurately. In the future, verification using saliency mapping is required to determine whether the insufficient accuracy in sub-step classification task was actually due to the similarity of image features between each sub-step.

In conclusion, the results of this study demonstrated that our deep learning model could be utilized to automatically identify steps of TaTME from an intraoperative video with a high degree of accuracy. However, our classification model needs to be trained with a larger dataset of intraoperative videos before it can be applied in practice.

## Supplementary Information

Below is the link to the electronic supplementary material.

Supplementary file1 (DOCX 174 KB)

## References

[1] Sylla P, Rattner DW, Delgado S, Lacy AM (2010). NOTES transanal rectal cancer resection using transanal endoscopic microsurgery and laparoscopic assistance. Surg Endosc.

[2] Targarona EM, Balague C, Pernas JC, Martinez C, Berindoague R, Gich I, Trias M (2008). Can we predict immediate outcome after laparoscopic rectal surgery? Multivariate analysis of clinical, anatomic, and pathologic features after 3-dimensional reconstruction of the pelvic anatomy. Ann Surg.

[3] Lacy AM, Tasende MM, Delgado S, Ferandez-Hevia M, Jimenez M, De Lacy B, Castells A, Bravo R, Wexner SD, Heald RJ (2015). Transanal total mesorectal excision for rectal cancer: outcomes after 140 patients. J Am Coll Surg.

[4] Rubinkiewicz M, Nowakowski M, Wierdak M, Mizera M, Dembiński M, Pisarska M, Major P, Małczak P, Budzyński A, Pędziwiatr M (2018). Transanal total mesorectal excision for low rectal cancer: a case-matched study comparing TaTME versus standard laparoscopic TME. Cancer Manag Res.

[5] Adamina M, Buchs NC, Penna M, Hompes R (2018). St Gallen Colorectal Consensus Expert Group. St Gallen consensus on safe implementation of transanal total mesorectal excision. Surg Endosc.

[6] Francis N, Penna M, Mackenzie H, Carter F, Hompes R (2017). Consensus on structured training curriculum for transanal total mesorectal excision (TaTME). Surg Endosc.

[7] Penna M, Hompes R, Arnold S, Wynn G, Austin R, Warusavitarne J, Moran B, Hanna GB, Mortensen NJ, Tekkis PP (2016). TaTME Registry Collaborative (2016) Transanal total mesorectal excision: international registry results of the first 720 cases. Ann Surg.

[8] Celentano V, Smart N, McGrath J, Cahill RA, Spinelli A, Obermair A, Hasegawa H, Lal P, Almoudaris AM, Hitchins CR, Pellino G (2018). LAP-VEGaS Practice Guidelines for reporting of educational videos in laparoscopic surgery: a joint trainers and trainees consensus statement. Ann Surg.

[9] Celentano V, Smart N, Cahill RA, McGrath JS, Gupta S, Griffith JP, Acheson AG, Cecil TD, Coleman MG (2019). Use of laparoscopic videos amongst surgical trainees in the United Kingdom. Surgeon.

[10] Foster JD, Miskovic D, Allison AS, Conti JA, Ockrim J, Cooper EJ, Hanna GB, Francis NK (2016). Application of objective clinical human reliability analysis (OCHRA) in assessment of technical performance in laparoscopic rectal cancer surgery. Tech Coloproctol.

[11] van Rutte P, Nienhuijs SW, Jakimowicz JJ, van Montfort G (2017). Identification of technical errors and hazard zones in sleeve gastrectomy using OCHRA: "OCHRA for sleeve gastrectomy". Surg Endosc.

[12] Tanaka R, DeAsis F, Vigneswaran Y, Linn J, Carbray J, Denham W, Haggerty S, Ujiki M (2018). Video review program enhances resident training in laparoscopic inguinal hernia: a randomized blinded controlled trial. Surg Endosc.

[13] Hamour AF, Mendez AI, Harris JR, Biron VL, Seikaly H, Côté DWJ (2018). A High-Definition Video Teaching Module for Thyroidectomy Surgery. J Surg Educ.

[14] Lecun Y, Bottou L, Bengio Y, Haffner P (1998). Gradient-based learning applied to document recognition. Proc IEEE.

[15] Hashimoto DA, Rosman G, Rus D, Meireles OR (2018). Artificial Intelligence in Surgery: Promises and Perils. Ann Surg.

[16] Twinanda AP, Shehata S, Mutter D, Marescaux J, de Mathelin M, Padoy N (2017). EndoNet: a deep architecture for recognition tasks on laparoscopic videos. IEEE Trans Med Imag.

[17] Nakawala H, Bianchi R, Pescatori LE, De Cobelli O, Ferrigno G, De Momi E (2019). "Deep-Onto" network for surgical workflow and context recognition. Int J Comput Assist Radiol Surg.

[18] Hashimoto DA, Rosman G, Witkowski ER, Stafford C, Navarette-Welton AJ, Rattner DW, Lillemoe KD, Rus DL, Meireles OR (2019). Computer vision analysis of intraoperative video: automated recognition of operative steps in laparoscopic sleeve gastrectomy. Ann Surg.

[19] Kitaguchi D, Takeshita N, Matsuzaki H, Takano H, Owada Y, Enomoto T, Oda T, Miura H, Yamanashi T, Watanabe M, Sato D, Sugomori Y, Hara S, Ito M (2019). Real-time automatic surgical phase recognition in laparoscopic sigmoidectomy using the convolutional neural network-based deep learning approach. Surg Endosc.

[20] Arroyave MC, DeLacy FB, Lacy AM (2017). Transanal total mesorectal excision (TaTME) for rectal cancer: step by step description of the surgical technique for a two-teams approach. Eur J Surg Oncol.

[21] Chollet F (2017). Xception: Deep Learning with depthwise separable convolutions. Proc IEEE Conf Comput Vis Pattern Recognit.

[22] Russakovsky O, Deng J, Su H, Krause J, Satheesh S, Ma S, Huang Z, Karpathy A, Khosla A, Bernstein M, Berg AC (2015). ImageNet large scale visual recognition challenge. Int J Comput Vision.

[23] Gholinejad M, Loeve AJ, Dankelman J (2019). Surgical process modeling strategies: which method to choose for determining workflow?. Minim Invasive Ther Allied Technol.

[24] Lessmann N, van Ginneken B, de Jong PA, Išgum I (2019). Iterative fully convolutional neural networks for automatic vertebra segmentation and identification. Med Image Anal.

[25] Fan J, Cao X, Yap PT, Shen D (2019). BIRNet: brain image registration using dual-supervised fully convolutional networks. Med Image Anal.

[26] Zhang X, Wang J, Wang T, Ji X, Shen Y, Sun Z, Zhang X (2019). A markerless automatic deformable registration framework for augmented reality navigation of laparoscopy partial nephrectomy. Int J Comput Assist Radiol Surg.

[27] Fujii K, Gras G, Salerno A, Yang GZ (2018). Gaze gesture based human robot interaction for laparoscopic surgery. Med Image Anal.

[28] Hong N, Kim M, Lee C, Kim S (2019). Head-mounted interface for intuitive vision control and continuous surgical operation in a surgical robot system. Med Biol Eng Comput.

[29] Padoy N, Blum T, Ahmadi SA, Feussner H, Berger MO, Navab N (2012). Statistical modeling and recognition of surgical workflow. Med Image Anal..

[30] Kranzfelder M, Schneider A, Fiolka A, Koller S, Reiser S, Vogel T, Wilhelm D, Feussner H (2014). Reliability of sensor-based real-time workflow recognition in laparoscopic cholecystectomy. Int J Comput Assist Radiol Surg.

[31] Dergachyova O, Bouget D, Huaulmé A, Morandi X, Jannin P (2016). Automatic data-driven real-time segmentation and recognition of surgical workflow. Int J Comput Assist Radiol Surg.

[32] Meeuwsen FC, van Luyn F, Blikkendaal MD, Jansen FW, van den Dobbelsteen JJ (2019). Surgical phase modeling in minimal invasive surgery. Surg Endosc.

[33] Veltcamp Helbach M, van Oostendorp SE, Koedam TWA, Knol JJ, Stockmann HBAC, Oosterling SJ, Vuylsteke RCLM, de Graaf EJR, Doornebosch PG, Hompes R, Bonjer HJ, Sietses C, Tuynman JB (2020). Structured training pathway and proctoring; multicenter results of the implementation of transanal total mesorectal excision (TaTME) in the Netherlands. Surg Endosc.

