# Supplementary figures and images for: Deep learning-based automatic surgical step recognition in intraoperative videos for transanal total mesorectal excision
Source: Surg Endosc. 2021 Apr 6;36(2):1143–51. doi: 10.1007/s00464-021-08381-6 (PMC8758657; doi:10.1007/s00464-021-08381-6)

**Appendix A:** The confusion matrix of the results for surgical step classification.


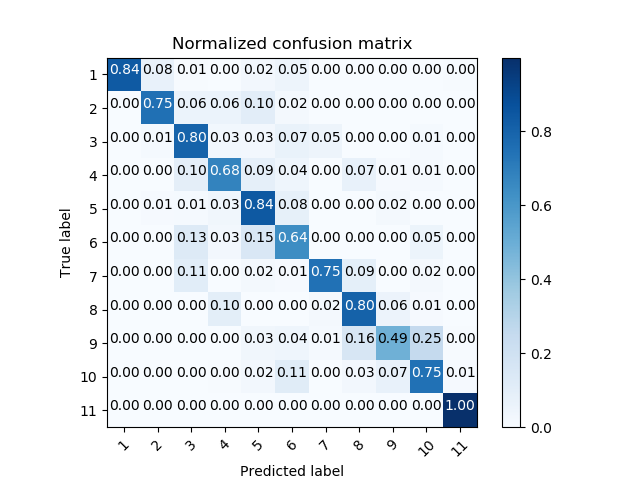

Supplement: Supplementary file 1 — Supplementary file1 (DOCX 174 KB) [file 464_2021_8381_MOESM1_ESM.docx]
